# Portal hypertension and hepatocellular carcinoma: Navigating uncharted waters

**DOI:** 10.1002/ueg2.12193

**Published:** 2022-01-08

**Authors:** Sabrina Sidali, Jean‐Charles Nault

**Affiliations:** ^1^ Université de Paris, Assistance‐Publique Hôpitaux de Paris, Hôpital Beaujon, Service d’Hépatologie, DMU DIGEST Clichy France; ^2^ Centre de Recherche des Cordeliers, Sorbonne Université, Inserm, Université de Paris, Team Functional Genomics of Solid Tumors, Equipe labellisée Ligue Nationale Contre le Cancer, Labex OncoImmunology Paris France; ^3^ Liver Unit, Hôpital Avicenne, Hôpitaux Universitaires Paris‐Seine‐Saint‐Denis, Assistance‐Publique Hôpitaux de Paris Bobigny France; ^4^ Unité de Formation et de Recherche Santé Médecine et Biologie Humaine, Université Paris 13, Communauté d’Universités et Etablissements Sorbonne Paris Cité Paris France

Portal hypertension (PHT) is the main determinant of liver cirrhosis decompensation, defined by the occurrence of ascites, variceal bleeding or hepatic encephalopathy.^1^, ^2^ A portal pressure gradient ≥10 mmHg, usually estimated by the hepatic venous pressure gradient (HVPG), defines clinically significant or evident PHT (clinically significant portal hypertension or clinically evident portal hypertension (CSPH or CEPH)), since liver decompensation might occur above this threshold.^3^ However, HVPG is not performed routinely in clinical practice, and the presence of esophageal/gastric varices and low platelet count associated with splenomegaly have been considered as surrogate markers of CEPH.^4^

Hepatocellular carcinoma (HCC) is another complication of cirrhosis and is closely linked to PHT. Hepatocellular carcinoma increases HVPG through the presence of arteriovenous shunting within the tumor and modifications of liver architecture whereas CEPH is predictive of HCC occurrence, independently of the severity of the underlying cirrhosis.^5^, ^6^ The occurrence of acute variceal bleeding and/or clinical ascites is also associated with poor prognosis in patients with HCC.^7^, ^8^ The impact of PHT in the treatment of patients with HCC was described more than 30 years ago when the Barcelona Clinic Liver Cancer team showed that presence of CEPH was associated with a high risk of postoperative hepatic decompensation and poor long term outcomes after liver resection.^9^ Consequently, current guidelines do not recommend hepatic resection for patients with CEPH.^10^, ^11^ Unfortunately, HCC is frequently diagnosed at intermediate and advanced stages when curative treatment cannot be applied. Trans arterial chemoembolization (TACE) is the treatment of choice in patients with intermediate‐stage HCC and may also be considered in selected patients with unresectable and not ablatable early stage HCC.^11^

For patients undergoing TACE, CEPH status has not been considered in the European Association for the Study of the Liver guidelines.^11^ However, TACE causes a decrease in hepatic arterial blood flow, accompanied by a transient increase in portal blood flow. The clinical implications of these changes in hepatic hemodynamics are unclear, with limited data on TACE outcomes in HCC patients with CEPH. Nevertheless, two retrospective, Korean studies in 2018 reported CEPH was associated with a decrease in overall survival (OS) after chemoembolization.^12^, ^13^

In the current issue of the *United European Gastroenterology Journal*, Müller et al. assess the prevalence and the prognostic impact of CEPH in a cohort of western patients with HCC that had undergone TACE.^14^ CEPH was defined by the presence of at least one of the following criteria: radiological ascites, esophageal/gastric varices, splenomegaly (>12 cm) and a low‐platelet count (<100 G/L). A total of 349 patients were included in the study of which 304 (87.1%) had cirrhosis. Among patients with cirrhosis, CEPH was observed in 241 (69.1%) patients. Median OS were 10.6 and 17.1 months in patients with and without CEPH, respectively (*p* = 0.036). In multivariate analysis, CEPH was not a significant risk factor for death (*p* = 0.190), and among the CEPH defining criteria, only ascites at imaging was associated with a lower OS (*p* < 0.003).

Müller et al. thus described some important observations. They reported that CEPH is present in more than two thirds of the patients with HCC that underwent TACE, in line with previous studies.^12^, ^13^ The absence of impact of CEPH on survival in multivariate analysis precludes its application to stratify use of TACE in intermediate HCC, but this finding should be interpreted with caution as OS was significantly impaired in univariate analysis. It could suggest that that the severity of CEPH could be more relevant than its presence by itself, as evidenced by the influence of ascites on outcomes. Furthermore, severe CEPH reflected by an inverse portal flow at ultrasonography is usually an exclusion criterion for TACE and it is unclear how many patients were excluded for this reason.

Overall, this study raises important questions in the field of HCC and PHT. Transjugular intrahepatic portosystemic shunt could be discussed in selected patients with small HCC and CEPH responsible of symptoms, in order to allow locoregional treatment or as a bridge to liver transplantation.^15^ The emergence of first‐line combined treatment using antiangiogenic (anti‐VEGF, bevacizumab) and anti‐PD‐L1 therapy (atezolizumab) as the gold standard in advanced HCC has led to new controversy on the risk of variceal bleeding due to antiangiogenic treatment. The modality of esophageal varices screening, choice of therapeutics for PHT and timing of introduction of the combined treatment in patients with PHT are still a matter of debate. Finally, more data in preclinical models and in real life are warranted to better decipher the complex interplay between PHT and outcomes after locoregional and systemic treatments in patients with HCC.

## CONFICT OF INTEREST

The authors have no conflict of interest to declare.

## Data Availability

No data, editorial.
